# Immediate Implant Placement With Peri-Implant Contour Augmentation in the Maxillary Central Incisor: A Three-Year Clinical and Cone-Beam Computed Tomography Follow-Up

**DOI:** 10.7759/cureus.117992

**Published:** 2026-10-15

**Authors:** B Tanuja, A Ramesh, Haritha Kotha, Y Pavani, Pocha Kalyani Rani

**Affiliations:** 1 Department of Periodontics, G. Pullareddy Dental College and Hospital, Kurnool, IND; 2 Department of Prosthodontics, G. Pullareddy Dental College and Hospital, Kurnool, IND

**Keywords:** alveolar bone loss, dental implants, guided bone regeneration, maxilla, osseointegration

## Abstract

Although immediate implant placement in the anterior maxilla can shorten the treatment timeline, it presents challenges in preserving peri-implant hard- and soft-tissue contours. We report the three-year clinical and radiographic outcome of immediate implant placement combined with peri-implant contour augmentation in the region of the maxillary right central incisor. A 20-year-old man presented two months after trauma with a non-restorable cervical fracture of the maxillary right central incisor. Clinical and cone-beam computed tomography (CBCT) evaluation demonstrated facial bone deficiency with sufficient apical and palatal bone for immediate implant placement. Following atraumatic extraction, a 4 × 14 mm implant was placed in a prosthetically driven three-dimensional position with primary stability of 35 Ncm. The peri-implant gap and facial contour were augmented using deproteinized bovine bone mineral (Bio-Oss®, Geistlich Pharma AG, Wolhusen, Switzerland) and a resorbable collagen membrane. Following uneventful healing, second-stage surgery was performed at three months, followed by definitive ceramic crown placement. At the three-year follow-up, the implant remained clinically stable, with healthy peri-implant soft tissues and preserved facial contour, without signs of inflammation, infection, or mobility. CBCT demonstrated maintained bone in the crestal and mid-buccal regions, with satisfactory osseointegration and preservation of peri-implant hard-tissue support. Careful case selection, atraumatic extraction, prosthetically driven implant positioning, primary stability, and appropriate management of facial bone deficiencies are important considerations for achieving predictable outcomes.

## Introduction

Immediate implant placement is a commonly adopted technique for dental rehabilitation in the anterior maxilla and can shorten the overall treatment sequence following extraction. However, placement of an implant immediately after extraction does not prevent the physiological remodeling of the extraction socket. The facial aspect of the alveolar ridge is particularly susceptible to dimensional alteration, which may affect the peri-implant tissue contour and the esthetic integration of the definitive restoration [1].

Achieving a predictable esthetic result in the anterior maxilla depends on the relationship between the implant and the surrounding hard and soft tissues. Facial bone thickness, implant position, gingival phenotype, interdental papillae, and prosthetically determined implant positioning are important considerations during treatment planning [2, 3]. In particular, a thin or deficient facial bone wall may increase the risk of facial tissue remodeling and mucosal recession. Recent evidence has demonstrated that immediate implant placement does not completely prevent buccal bone resorption and that the extent of facial bone changes can be influenced by the characteristics of the extraction site and the treatment protocol [4].

When a facial bone deficiency or a substantial peri-implant gap is present, simultaneous hard-tissue augmentation may be considered to support the facial contour. Deproteinized bovine bone mineral (DBBM), including Bio-Oss® (Geistlich Pharma AG, Wolhusen, Switzerland), has been widely used for peri-implant grafting because of its osteoconductive properties and slow resorption. Experimental and clinical studies have reported that grafting the peri-implant gap during immediate implant placement may reduce horizontal buccal bone dimensional changes compared with nongrafted sites [5, 6]. Clinical evidence involving immediate implants in the anterior maxilla with thin buccal plates has also demonstrated that simultaneous augmentation using DBBM and a resorbable membrane can help maintain buccal bone dimensions during healing [7]. Guided bone regeneration using a barrier membrane can additionally help stabilize the grafted area when a localized facial osseous defect is encountered. Evidence from contemporary systematic reviews indicates that regenerative procedures performed simultaneously with implant placement can provide predictable bone regeneration when appropriate principles of defect management, graft stabilization, and soft-tissue closure are followed [8, 9].

Nevertheless, the extent of benefit varies according to the initial defect, implant position, surgical technique, and patient-related factors. Although immediate implant placement with simultaneous augmentation has been extensively described, reports documenting the long-term clinical and radiographic outcome of contour augmentation in the presence of a facial bone deficiency remain clinically useful. The present case describes immediate implant placement in the maxillary right central incisor region following traumatic tooth fracture, with simultaneous Bio-Oss® and collagen membrane contour augmentation, and presents the clinical and CBCT findings after three years of follow-up.

## Case presentation

A 20-year-old man presented to the dental department for rehabilitation of a fractured maxillary anterior tooth. The patient had a history of trauma to the maxillary anterior region approximately two months prior to presentation. Following the trauma, the maxillary right central incisor (tooth 11) had sustained a fracture; however, the patient reported to the dental department only after an interval of approximately two months, seeking definitive replacement of the tooth. Clinical examination revealed a fracture involving the cervical third of the maxillary right central incisor (tooth 11), with extension below the gingival margin, rendering the tooth non-restorable. Based on the classification of traumatic dental injuries proposed by Andreasen, the injury was categorized as Class VI [10]. The adjacent teeth 41 and 42, which also showed signs of trauma, were managed with composite restorations, and the surrounding periodontal tissues were clinically healthy.

Considering the extent of the fracture and the poor prognosis of tooth 11, the patient was referred to the Department of Periodontology for further evaluation and management. Various treatment options for replacement of the tooth were discussed with the patient, including extraction followed by delayed implant placement and immediate implant placement. Following clinical and radiographic assessment, immediate implant placement following atraumatic extraction was planned to replace tooth 11. The facial gingival phenotype was clinically assessed as thick, with intact interdental papillae and an adequate zone of keratinized tissue. A periapical radiograph and cone-beam computed tomography (CBCT) scan were obtained to evaluate the residual tooth structure, surrounding alveolar bone, socket anatomy, and the available apical and palatal bone. CBCT examination demonstrated a deficient facial bone plate, while adequate apical and palatal bone was available for immediate implant placement (Figure 1b).

**Figure 1:**
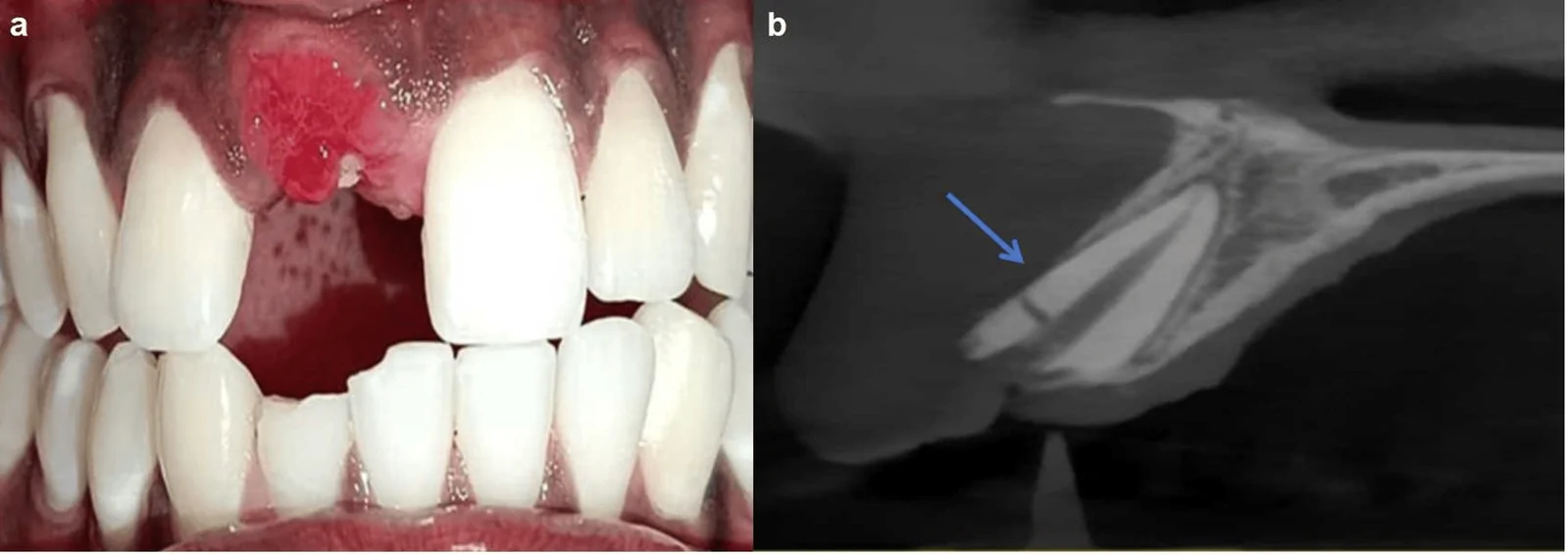
(a) Traumatic crown fracture involving the cervical region of tooth 11; (b) cone-beam computed tomography (CBCT) image showing a deficient facial bone plate.

The treatment plan, including atraumatic extraction, immediate implant placement, and simultaneous peri-implant contour augmentation using deproteinized bovine bone mineral (Bio-Oss®) with a collagen membrane, was explained to the patient. The potential benefits, limitations, and alternative treatment options were discussed, and written informed consent was obtained before treatment.

Surgical procedure

Following administration of local anesthesia, an intrasulcular incision was made circumferentially around tooth 11 using a 15C blade. The tooth was atraumatically extracted using an anterior periotome and appropriate extraction forceps, with an emphasis on preserving the socket walls. The extraction socket was subsequently debrided and inspected, and the integrity of the facial socket wall was clinically assessed (Figure 2a). A full-thickness mucoperiosteal flap was then reflected (Figure 2b), and sequential osteotomy preparation was performed. A 4 × 14 mm Dentium SuperLine implant with a sandblasted, large-grit, acid-etched (SLA) surface (Dentium Co., Seoul, Republic of Korea) was placed in a prosthetically driven three-dimensional position. Primary stability of 35 Ncm was achieved. The implant was positioned slightly palatally to maintain an appropriate facial contour and adequate distance from the adjacent teeth.

**Figure 2:**
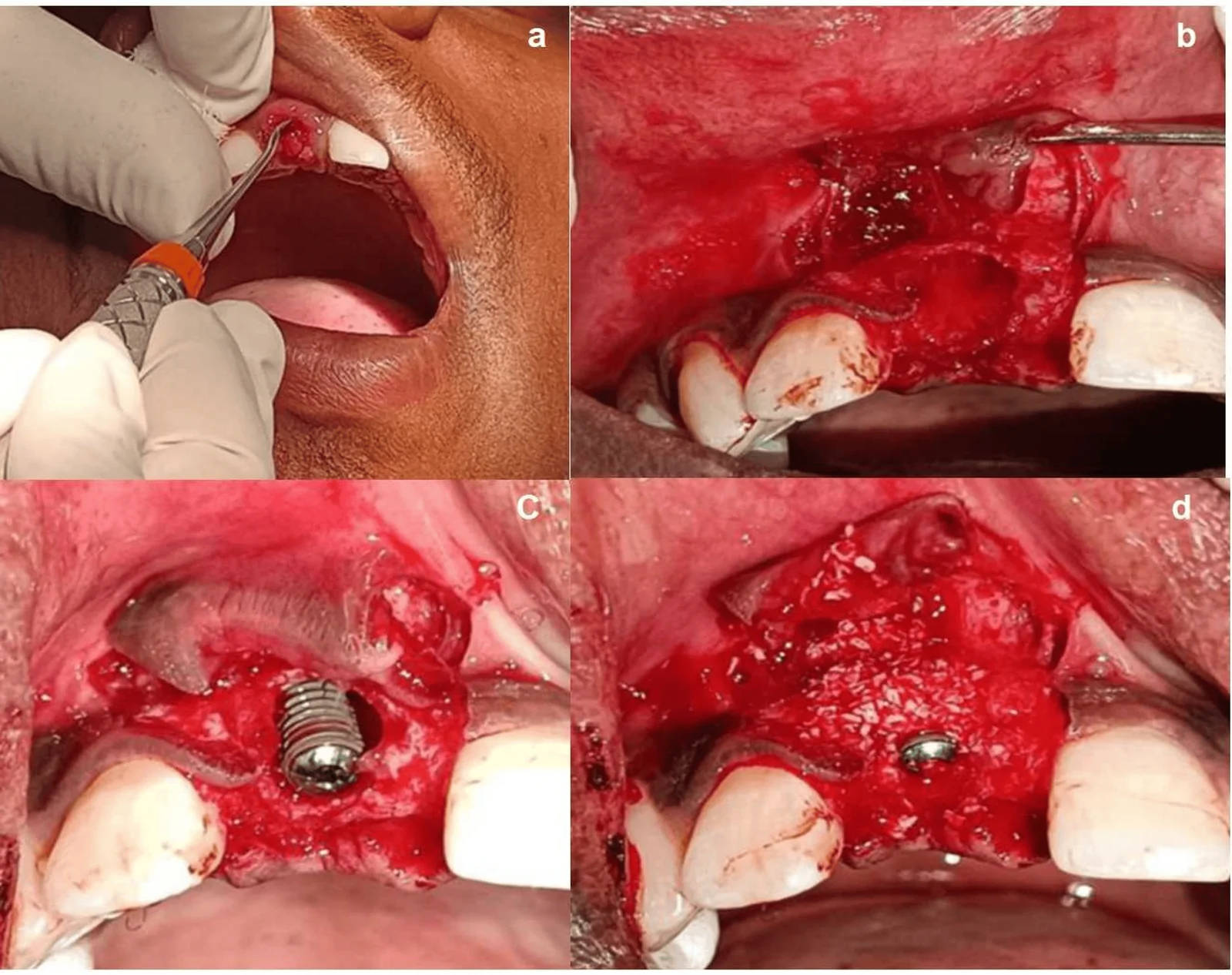
(a) Thoroughly debrided extraction socket; (b) full-thickness mucoperiosteal flap reflected, followed by sequential osteotomy preparation at the implant site; (c) partial exposure of the implant threads at the mid-buccal aspect; (d) placement of Bio-Oss® graft material around the implant and facial contour.

Following implant placement, partial exposure of the implant threads was observed at the mid-buccal aspect of the edentulous ridge (Figure 2c), corresponding to a localized buccal peri-implant osseous deficiency. The peri-implant gap and deficient facial contour were augmented with deproteinized bovine bone mineral (Bio-Oss®). A resorbable Bio-Gide® collagen membrane (Geistlich Pharma AG, Switzerland) was placed over the grafted area (Figure 2d). A cover screw was placed, and the flap was repositioned and primarily closed. Because adequate primary stability of 35 Ncm was achieved, no provisional restoration was provided, and the implant was allowed to heal in a submerged manner for three months. Postoperative instructions were provided, and the patient was scheduled for follow-up.

Follow-up and clinical and radiographic evaluation

At the two-week postoperative review, healing was uneventful, and the sutures were removed. After three months, second-stage surgery was performed, and a healing abutment was placed. Two weeks after placement of the healing abutment, a digital impression was obtained, and the definitive ceramic crown was delivered. The restoration was adjusted to establish appropriate occlusion, proximal contacts, emergence profile, and esthetic integration with the adjacent dentition. The patient was scheduled to follow up at six, 12, 18, 24, and 36 months.

 At the six- and 12-month follow-up, the peri-implant soft tissues appeared clinically healthy, with no signs of inflammation, infection, or implant mobility. Probing depth was approximately 4 mm, with no bleeding on probing, and good oral hygiene was maintained. The esthetic outcome was assessed using the Pink Esthetic Score (PES) described by Fürhauser et al. [11] and the White Esthetic Score (WES) described by Belser et al. [12], with scores of 12/14 and 8/10, respectively (Figure 3a). CBCT evaluation at 12 months demonstrated approximately 2.2 mm of facial bone thickness, measured at a reference level 2 mm apical to the implant platform (Figure 3b). CBCT imaging was performed using a CS 9600 CBCT unit (Carestream Dental, Atlanta, GA, USA) with imaging parameters of 120 kVp, 3.8 mA, an exposure time of <12 seconds, and a 5 × 5 cm field of view. The same slice orientation was used for the one- and three-year assessments.

**Figure 3:**
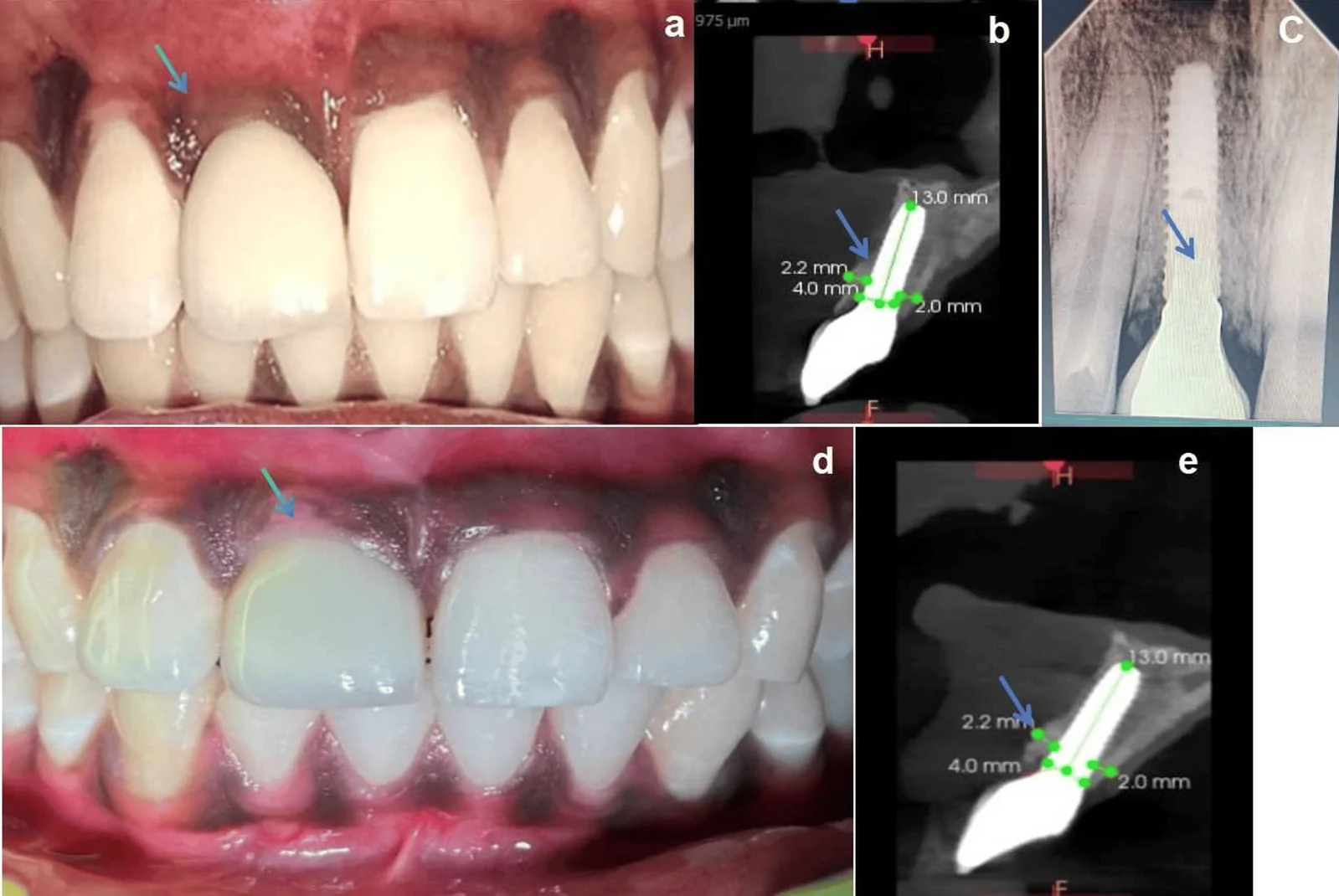
Follow-up clinical and radiographic images. (a) Clinical appearance at the 12-month follow-up; (b) CBCT evaluation at the 12-month follow-up showing approximately 2.2 mm of facial bone thickness; (c) IOPA evaluation at the 24-month follow-up; (d) clinical appearance at the three-year follow-up; (e) CBCT evaluation at the three-year follow-up showing maintenance of the facial bone without evident radiographic resorption.

The patient did not attend the planned 18-month follow-up but returned at approximately 24 months, when an intraoral radiographic (RVG) assessment was obtained (Figure 3c). The patient subsequently returned for the three-year clinical and radiographic evaluation. At three years, the peri-implant soft tissues remained clinically healthy, with no bleeding on probing, a probing depth of approximately 4 mm, and no clinical signs of inflammation, infection, or implant mobility. The PES and WES remained unchanged at 12/14 and 8/10, respectively (Figure 3d). The three-year CBCT demonstrated maintenance of the approximately 2.2-mm facial bone thickness observed at one year, without evident radiographic resorption. The crestal and mid-buccal bone were preserved, with satisfactory radiographic evidence of implant osseointegration and maintained peri-implant hard-tissue support (Figure 3e).

## Discussion

Immediate implant placement is an established treatment approach for replacement of teeth in the anterior maxilla, particularly when preservation of the peri-implant soft-tissue architecture and reduction of treatment time are important considerations. However, immediate implant placement does not eliminate the physiological remodeling that follows tooth extraction. Buccal bone resorption and dimensional alteration of the extraction socket may occur even when an implant is placed immediately, making management of the facial aspect particularly important in the esthetic zone. A systematic review and meta-analysis reported mean horizontal and vertical buccal bone dimensional changes of approximately 0.71 mm and 0.58 mm, respectively, following immediate implant placement, indicating that immediate placement alone does not completely prevent post-extraction remodeling [4].

The present case involved a young patient with a traumatized maxillary right central incisor that had a fracture extending to the cervical one-third and was considered to have a poor prognosis for preservation. Following clinical and CBCT assessment, immediate implant placement was selected after atraumatic extraction. An important finding in this case was the preoperative facial bone deficiency, which represented a potential challenge for maintenance of the facial peri-implant contour. The anterior maxilla is particularly sensitive to small changes in hard- and soft-tissue dimensions because the facial tissues contribute substantially to the esthetic outcome. Recent systematic evidence indicates that a thinner facial bone wall is associated with a greater risk of gingival recession or facial tissue shrinkage following immediate implant treatment [13]. Therefore, assessment of the facial bone and careful three-dimensional implant positioning are important components of treatment planning.

The implant was placed palatally with primary stability of 35 Ncm, enabling prosthetically driven three-dimensional positioning while preserving alignment with adjacent teeth and the facial contour. However, after implant placement, partial exposure of the implant threads was observed at the mid-buccal aspect, confirming a localized facial osseous deficiency. Simultaneous contour augmentation was performed with deproteinized bovine bone mineral (Bio-Oss®), followed by placement of a resorbable collagen membrane to manage the defect.

Clinical evidence supports grafting peri-implant gaps, with a systematic review and meta-analysis of randomized controlled trials showing significantly reduced horizontal buccal bone resorption and less apical migration of the mid-facial soft-tissue level in grafted sites, though data on other esthetic and periodontal outcomes remain limited [6]. These findings support the use of grafting as an adjunct in selected immediate implant cases, particularly when preservation of the facial contour is a clinical objective.

The use of deproteinized bovine bone mineral with a membrane in the present case is also consistent with clinical evidence from the anterior maxilla. Chen et al. reported that immediate implant placement with buccal bone augmentation using deproteinized bovine bone mineral and an absorbable membrane maintained buccal bone dimensions at one year, though some reduction in thickness occurred during healing [7]. Similarly, a recent systematic review and meta-analysis concluded that hard- and/or soft-tissue grafting associated with immediate implant placement may contribute to improved facial bone thickness and peri-implant tissue stability, although significant differences were not demonstrated for all esthetic parameters, including the Pink Esthetic Score [8].

The collagen membrane used in the present case served as a barrier over the grafted region. The principle of guided bone regeneration is particularly relevant when a localized buccal dehiscence or contour deficiency is encountered around an implant. A network meta-analysis of guided bone regeneration procedures performed simultaneously with implant placement reported that bone regeneration can be achieved predictably when appropriate regenerative principles are followed, irrespective of the specific biomaterial used. However, some degree of resorption of augmented bone may occur during follow-up, and membrane-related complications such as exposure, infection, and soft-tissue dehiscence have been reported [9]. In the present case, no such postoperative complication was documented, and primary soft-tissue closure was maintained.

Soft-tissue phenotype is another important factor in immediate implant therapy. The patient in this case presented with a thick facial gingival phenotype, intact interdental papillae, and an adequate zone of keratinized tissue. These characteristics may have contributed to favorable soft-tissue maintenance; however, the outcome cannot be attributed to gingival phenotype alone because multiple treatment-related factors, including implant position, facial bone management, grafting, membrane placement, and prosthetic contour, were involved. Systematic evidence has shown that connective tissue grafting can improve midfacial soft-tissue stability in immediate implant therapy, particularly in patients with a thin phenotype or limited facial bone thickness [14]. Conversely, long-term clinical evidence indicates that favorable esthetic outcomes can also be obtained without connective tissue grafting in appropriately selected cases. Thus, soft-tissue augmentation should be considered according to the individual anatomical and esthetic risk profile rather than as a mandatory component of every immediate implant procedure.

An important feature of the present case is the three-year follow-up. The patient demonstrated clinically healthy peri-implant soft tissues, absence of implant mobility or clinical signs of inflammation, and satisfactory esthetic integration of the definitive ceramic restoration. The three-year CBCT examination demonstrated bone formation in the crestal and mid-buccal regions with satisfactory radiographic evidence of osseointegration. Long-term follow-up is particularly relevant because early postoperative stability does not necessarily indicate preservation of the peri-implant tissues over time. Although most studies evaluating grafting around immediate implants have relatively short follow-up periods, available long-term evidence supports the potential for stable clinical and esthetic outcomes in appropriately selected patients.

An important finding in the present case was the maintenance of approximately 2.2 mm of facial bone at the one- and three-year follow-ups, with no evident radiographic resorption during the observation period. This suggests that the combined use of deproteinized bovine bone mineral and a collagen membrane may have contributed to the preservation of the peri-implant facial hard-tissue contour following immediate implant placement. The stable crestal and mid-buccal bone observed at three years, together with satisfactory osseointegration and healthy peri-implant soft tissues, indicates a favorable long-term tissue response in this case. The favorable outcome observed in this patient should nevertheless be interpreted cautiously. This was a single case involving a young patient with a thick gingival phenotype, clinically healthy adjacent tissues, adequate apical and palatal bone, and successful primary implant stability. Furthermore, several interventions were performed simultaneously, including atraumatic extraction, prosthetically driven implant positioning, peri-implant bone grafting, collagen membrane placement, and subsequent prosthetic rehabilitation. Therefore, the three-year outcome cannot be attributed exclusively to Bio-Oss® contour augmentation, nor can the findings be generalized to all immediate implant cases.

The clinical significance of this case lies in the management of a localized facial bone deficiency encountered during immediate implant placement in the esthetic zone and the documented three-year clinical and CBCT follow-up. The case illustrates that careful preoperative three-dimensional assessment, atraumatic extraction, appropriate implant positioning, and simultaneous management of a facial peri-implant defect can be incorporated into a comprehensive immediate implant protocol. Further controlled clinical studies with standardized CBCT assessments and extended follow-up are necessary to establish the contribution of contour augmentation to the long-term preservation of peri-implant hard and soft tissues. In addition, CBCT assessment of thin facial peri-implant bone may be affected by titanium-related artifacts, which represents a further limitation of radiographic interpretation.

## Conclusions

Immediate implant placement combined with peri-implant contour augmentation using deproteinized bovine bone mineral (Bio-Oss®) and a collagen membrane achieved favorable clinical and esthetic outcomes over three years, emphasizing the importance of case selection, atraumatic extraction, prosthetically guided positioning, primary stability, and management of facial bone deficiencies in the anterior maxilla. The stable clinical and CBCT findings at three years support the potential value of this approach in selected cases; however, the results of a single case should not be generalized to all immediate implant procedures.
